# Effects of Exercise-Based Interventions on Musculoskeletal Pain and Physical Function in Manufacturing Workers: A Systematic Review and Meta-Analysis

**DOI:** 10.3390/healthcare14193230

**Published:** 2026-10-01

**Authors:** KiHyun Kim, HyungSoo Shin, HeeJoon Shin, WanSuk Choi, MiRan Goo, KyuRyeong Kim, MinBong Kang

**Affiliations:** 1 Department of Physical Therapy, KyungWoon University, Gumi 39160, Republic of Korea; khpt05@ikw.ac.kr (K.K.); lnsshj@ikw.ac.kr (H.S.); wschoi@ikw.ac.kr (W.C.);; 2 Department of Physical Therapy, Daegu Medical Foundation K Hospital, Daegu 41452, Republic of Korea; 3 Department of Physical Therapy, Daejeon Institute of Science and Technology, Daejeon 35408, Republic of Korea

**Keywords:** workplace exercise, work-related musculoskeletal disorders, manufacturing workers, occupational health, systematic review, meta-analysis

## Abstract

**Highlights:**

**What are the main findings?**

- The pooled pain estimate favored exercise-based interventions, but substantial heterogeneity and very-low-certainty evidence limit confidence in this finding.
- Evidence certainty was low for disability/function-status outcomes and very low for shoulder range of motion and RAND-36 physical functioning; the confidence intervals for all three functional domains included no effect.

**What are the implications of the main findings?**

- Substantial heterogeneity in pain outcomes and limited evidence certainty warrant caution when applying the pooled findings to specific manufacturing settings.
- The optimal exercise type, intensity, frequency, and duration for specific manufacturing tasks and musculoskeletal conditions remain uncertain; further high-quality trials are needed to clarify these prescriptions and their long-term effects.

**Abstract:**

**Background/Objectives**: Work-related musculoskeletal disorders (WMSDs) are prevalent among manufacturing workers exposed to repetitive and physically demanding tasks. This systematic review and meta-analysis evaluated the effects of exercise-based interventions on musculoskeletal pain and physical function among manufacturing workers, focusing on symptom management and functional improvement rather than the prevention of new WMSDs. **Methods**: PubMed, Cochrane Library, Embase, CINAHL, and Web of Science were searched from inception to March 31, 2026. Prospective controlled studies of workplace- or job-related exercise interventions were included. Risk of bias was evaluated within the RoB 2 framework, and random-effects meta-analyses used standardized mean differences (Hedges’ g), with functional outcomes analyzed separately by domain. The review was registered in PROSPERO (CRD420261369113). **Results:** Thirteen studies were included in the review. The revised primary pain synthesis included seven studies (414 participants) and favored exercise-based interventions (SMD −0.69, 95% CI −1.25 to −0.12; I^2^ = 71.9%). Disability/function-status outcomes included three studies (143 participants; SMD −0.67, 95% CI −1.42 to 0.07). Estimates for shoulder abduction range of motion (two studies; 111 participants; SMD −0.63, 95% CI −3.29 to 2.03) and RAND-36 physical functioning (one study; 24 participants; SMD −0.21, 95% CI −1.06 to 0.64) were imprecise. Evidence certainty was low for disability/function-status outcomes and very low for the other outcomes. **Conclusions:** Exercise-based interventions may improve disability/function-status outcomes, although the confidence interval included little or no difference. Evidence for effects on pain, shoulder range of motion, and RAND-36 physical functioning remains very uncertain. The findings concern symptom management rather than demonstrated prevention of new WMSDs. Optimal exercise prescriptions and sustained benefits remain uncertain.

## 1. Introduction

Work-Related Musculoskeletal Disorders (WMSDs) are a significant global occupational disease that reduces workers’ functional abilities and causes temporary or permanent work impairment, resulting in a substantial health and economic burden [1]. The prevalence of musculoskeletal disorders is particularly high among workers in industries and manufacturing that have high physical and mental workloads, due to excessive physical demands and repetitive manual labor [2]. These workers experience pain in various parts of the body, including the wrists, neck, shoulders, and lower back. This pain demonstrates a statistically significant strong positive correlation with the overall physical load indicators in the workplace [3,4].

Manufacturing workers perform tasks involving repetitive movements, forceful exertion, and sustained or awkward postures, making occupational demands central to the interpretation of musculoskeletal outcomes [2,3,4]. Previous reviews have examined exercise interventions among manual workers and workplace interventions among employees with physically demanding work [5,6]. However, the applicability of these broader findings to specific manufacturing settings requires consideration of differences in occupational tasks, musculoskeletal conditions, and intervention content.

Exercise-based programs incorporating flexibility, strength, and balance have been proposed as workplace interventions to address musculoskeletal health [7]. Previous studies have reported improvements in musculoskeletal pain following workplace exercise interventions [8,9]. However, improvements in symptoms or function should be distinguished from the prevention of new WMSDs.

A manufacturing-focused synthesis is needed to clarify the effects of exercise-based interventions on musculoskeletal pain and physical function and the extent to which these findings can inform occupational rehabilitation. Accordingly, this review aimed to synthesize controlled studies evaluating these outcomes among manufacturing workers and to assess the certainty and applicability of the evidence. The review focuses on pain and functional outcomes rather than the incidence of new WMSDs and therefore does not establish the effectiveness of exercise for primary prevention.

## 2. Materials and Methods

### 2.1. Study Screening and Data Collection

The present study was conducted in accordance with the guidelines for systematic reviews and meta-analyses (PRISMA 2020) and was pre-registered with PROSPERO (CRD420261369113). The completed PRISMA 2020 checklist is provided in Supplementary S8. As this study involved secondary analysis of previously published data only, IRB approval was not required. Five electronic databases were searched: PubMed, Cochrane Library, Embase, CINAHL, and Web of Science. The search covered each database from inception through 31 March 2026, without language restrictions. The search concepts addressed manufacturing or industrial workers, musculoskeletal disorders or pain, exercise interventions, and controlled trials. No dedicated searches of grey literature sources, trial registries, dissertation repositories, or conference proceedings were conducted beyond the five databases. Eligible studies were restricted to peer-reviewed prospective controlled studies. The search strategies are presented in Supplementary Material S1. The selection of studies was conducted in accordance with the PICOS criteria.

Eligible participants were adult workers employed in the manufacturing sector, including factories, assembly lines, and production jobs. An established WMSD diagnosis was not required for inclusion. Studies involving workers with existing musculoskeletal symptoms or diagnosed conditions, as well as studies involving workers at risk of WMSDs in manufacturing settings, were eligible. Thus, eligibility was not restricted to either a treatment population or a population without existing symptoms. The synthesis focused on pain and physical-function outcomes rather than the incidence of new WMSDs. Eligible interventions were workplace- or job-related programs lasting at least two weeks that included physical exercise, such as stretching, strengthening, resistance training, or active range-of-motion exercises. The two-week minimum was an eligibility restriction applied in this review and should not be interpreted as an established minimum duration required for clinical benefit. Programs combining exercise with non-exercise components, such as ergonomic education, manual therapy, massage, or physical agent modalities, were also eligible. Accordingly, “exercise-based” denotes the inclusion of an exercise component and does not imply that exercise was the sole component of the intervention. Eligible comparators included no additional intervention or usual work activities, waitlist controls, ergonomic education or training without the exercise program under evaluation, and active non-exercise treatments such as paraffin therapy or conventional physical therapy. Comparisons in which both groups received the same non-exercise treatment and exercise was added only to the intervention group were also included. Eligible outcomes included pain intensity, assessed using instruments such as the visual analog scale (VAS) or numerical rating scale (NRS), and measures related to physical functioning. The eligibility definition for physical functioning was broad and encompassed range of motion, muscle strength, and disability or functional-status measures. These categories represent different aspects of musculoskeletal health, including physical impairments and limitations in activities, and should not be regarded as interchangeable measures of a single construct. The study design was limited to peer-reviewed prospective controlled studies (RCTs and CCTs). Studies exclusively focusing on office workers or service industry employees, environmental improvement studies without physical exercise, and single-group studies without a control group were excluded.

Two reviewers independently screened titles and abstracts of all retrieved records against the eligibility criteria. Full-text articles of potentially eligible studies were then assessed independently by the same two reviewers. Discrepancies were resolved through discussion and, when necessary, by consultation with a third reviewer. Data extraction was performed independently by two reviewers using a standardized data extraction form. Extracted data included study characteristics (author, year, country, study design), participant characteristics (sample size, age, sex), intervention details (type, frequency, duration, volume), control group details, and outcome measures. Any disagreements were resolved by consensus or by a third reviewer.

### 2.2. Risk of Bias Assessment

The qualitative evaluation of the included studies was performed by two independent researchers. For individually randomized controlled trials, the Cochrane Risk of Bias 2 (RoB 2) tool was used. Its five domains are bias arising from the randomization process, bias due to deviations from intended interventions, bias due to missing outcome data, bias in measurement of the outcome, and bias in selection of the reported result. For Gundmi et al. [10], the reported RoB 2 assessment included the additional cluster-specific domain concerning the timing of identification and recruitment of individual participants in relation to randomization (Supplementary S4). Re-examination of Lowe et al. [11] identified work-group randomization, making the previous ROBINS-I assessment inappropriate. Its methodological limitations were therefore described narratively; a complete assessment using the cluster-randomized version of RoB 2 was not available for the revised review. In instances of evaluator disagreement, a third researcher was consulted to reach a consensus decision.

### 2.3. Certainty of Evidence Assessment

The certainty of evidence for each primary outcome was assessed using the Grading of Recommendations, Assessment, Development and Evaluations (GRADE) approach. Evidence from RCTs begins at high certainty and may be downgraded based on five domains: risk of bias, inconsistency, indirectness, imprecision, and publication bias.

### 2.4. Calculation of Meta-Effects

During revision, we re-examined the aggregate data used in the quantitative synthesis and revised the analyses to address data-extraction and outcome-comparability concerns. These analytical changes were made during revision and were not prespecified. The revised analyses were implemented in Python (version 3.13.15) using NumPy (version 2.1.3) and SciPy (version 1.16.3). Between-group effects were calculated from changes from baseline and expressed as standardized mean differences using Hedges’ g. For outcomes in which lower scores indicate improvement, changes were calculated as follow-up minus baseline. For outcomes in which higher scores indicate improvement, the direction was reversed so that negative SMDs consistently favoured the exercise-based intervention. Directly reported change-score means and standard deviations were used when they could be matched to the selected outcome, assessment time point, and participant group. When change-score standard deviations were not directly available, they were reconstructed from reported within-group statistics where possible. Otherwise, they were estimated from baseline and follow-up standard deviations assuming a within-person correlation of 0.50. The calculation was SD_change = √(SD_baseline^2^ + SD_follow-up^2^ − 2r × SD_baseline × SD_follow-up), where r denotes the assumed within-person correlation between baseline and follow-up measurements. Discrepancies between tables were documented, and incompatible summary statistics were not combined within a single effect-size calculation. Standardization addresses differences in measurement units but does not establish that different instruments assess the same clinical construct.

In the revised quantitative synthesis, functional outcomes were grouped into three domains: disability/activity limitation, comprising the physical-function subscale of the Australian/Canadian Osteoarthritis Hand Index (AUSCAN), the Roland–Morris Disability Questionnaire (RMDQ), and the Oswestry Disability Index (ODI); shoulder abduction range of motion; and the physical-functioning scale of the RAND 36-Item Health Survey (RAND-36). No overall effect was pooled across these domains. Each study contributed only one comparison to each outcome-domain synthesis. The RAND-36 physical-functioning result was reported as a single-study estimate. These domain groupings were introduced during revision and were not prespecified.

Sample sizes were aligned with the selected measurement occasion. For Nassif et al. [12], the analysis used outcomes assessed at completion of the two-month intervention, with 32 intervention and 28 control participants. The six-month follow-up data were not included in these comparisons. For Pehlevan and Şevgin [13], the directly reported change-score means and standard deviations were used. For Park et al. [14], the lumbar stabilization and Nintendo Wii exercise groups were combined into a single exercise group using sample-size-weighted means and a combined variance accounting for differences between the group means. The combined exercise group included 16 participants, and the shared control group of eight participants was included once in each relevant analysis.

For Kang et al. [15], directly reported pain change scores were extracted from Table 4 of the source report.

For the AUSCAN physical-function subscale, inconsistencies between Tables 2 and 4 of Kang et al. [15] prevented unambiguous use of the change-score row labels in Table 4 of that report. We therefore used the physical-function means and within-group t statistics in Table 2 of the same report to reconstruct the change-score standard deviations as SD_change = |mean change| × √*n*/|t|. The reconstructed standard deviations were approximately 10.21 and 7.19 for the intervention and control groups, respectively.

For Rasotto et al. [16], the primary pain and shoulder range-of-motion calculations used baseline and 10-month final-assessment means from Table 3, with change-score standard deviations estimated using the assumed baseline–follow-up correlation. The intermediate five-month assessment was not included as an additional comparison. Because these changes differed from the change statistics reported in Table 4, sensitivity analyses used the alternative Table 4 statistics. An additional pain analysis excluded this study. These analyses evaluated the consequences of the discrepancy without establishing which set of source statistics was correct.

For descriptive purposes, intervention composition was classified according to the full program delivered to the intervention group. Single-component interventions comprised exercise without an additional non-exercise intervention; different exercise modalities, such as stretching and strengthening, were treated as components of the exercise program rather than as separate treatment categories. Multicomponent interventions combined exercise with one or more non-exercise interventions, such as education, manual therapy, massage, or physical agent modalities. This classification included non-exercise interventions delivered to both groups as part of the intervention-group program. However, shared co-interventions were distinguished from components delivered exclusively to the intervention group when interpreting the treatment contrast. Treatments delivered only to the control group did not determine intervention classification.

Random-effects models were fitted using restricted maximum likelihood to estimate between-study variance (τ^2^). Confidence intervals and tests for pooled effects used a modified Hartung–Knapp approach, with the variance scaling factor constrained to be at least 1 and a t distribution with k−1 degrees of freedom, where k denotes the number of studies. Individual-study confidence intervals were calculated using a normal approximation. Statistical heterogeneity was described using Cochran’s Q, I^2^, and τ^2^.

Sensitivity analyses examined the influence of assuming baseline–follow-up correlations of 0.25 and 0.75 for outcomes with imputed change-score standard deviations. Leave-one-out analyses were conducted using the same REML framework. An additional sensitivity analysis examined the inclusion of the approximate data from Moreira-Silva et al. [17] in the pain synthesis. Lowe et al. [11] was retained in the review but excluded from the primary change-score meta-analysis. This study randomized at the work-group level and reported averages across repeated monthly assessments. The initial questionnaire respondent counts could not be confirmed as the denominators for paired change-score data, and a compatible effect estimate with an appropriate variance accounting for the study design could not be established from the available report.

Following revision of the quantitative datasets, formal meta-regression, intervention-complexity subgroup comparisons, funnel-plot asymmetry testing, and trim-and-fill analyses were not retained in the revised synthesis. The small number of studies within each revised outcome domain limited the informativeness of these analyses. Publication bias could not be excluded.

## 3. Results

### 3.1. Included Studies and Characteristics

**Figure 1:**
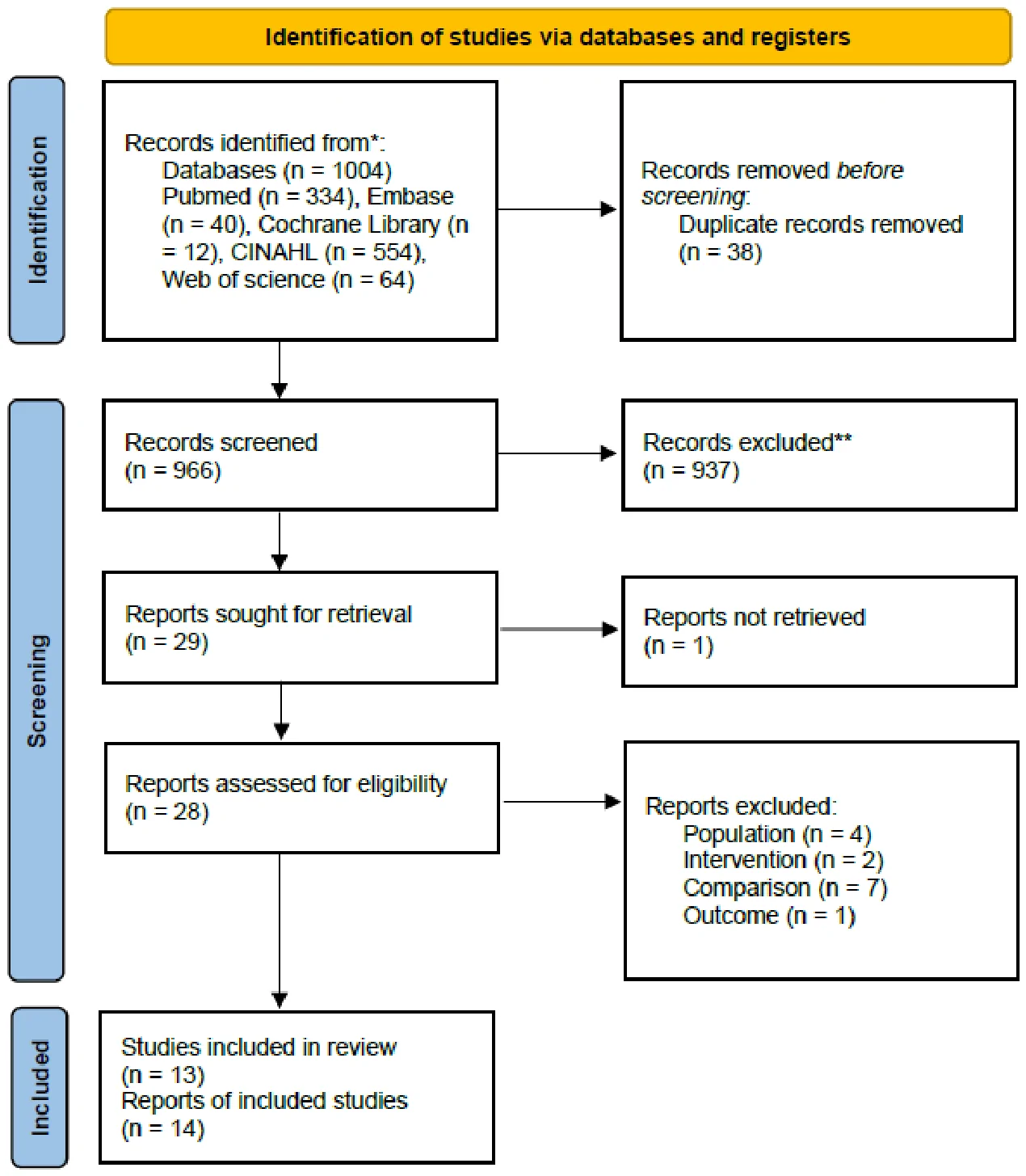
PRISMA Flow Diagram of the Study Selection Process. * Records identified from PubMed, Embase, the Cochrane Library, CINAHL, and Web of Science; counts for each database are shown in the figure. ** Records excluded during title and abstract screening.

A total of 1004 records were identified across the five databases, and 38 duplicate records were removed. The remaining 966 records underwent title and abstract screening, during which 937 were excluded. Full-text retrieval was sought for 29 reports; one report could not be retrieved, leaving 28 reports for full-text eligibility assessment. Of these, 14 were excluded because they did not meet the eligibility criteria for the population (*n* = 4), intervention (*n* = 2), comparator (*n* = 7), or outcome (*n* = 1). The remaining 14 reports represented 13 studies and were included in the review [10,11,12,13,14,15,16,17,18,19,20,21,22,23] (Figure 1; Table 1).

Two reports by Sundstrup et al. [22,23] described different outcomes from the same randomized trial of 66 slaughterhouse workers allocated to strength training or ergonomic training. These reports were counted as one study. In the revised synthesis, the pain findings from the 2016 report and the work-ability findings from the 2014 report were retained in the narrative synthesis rather than entered into the primary pooled analyses. The two reports were not treated as independent trials.

The 13 studies included in the review were prospective controlled studies involving manufacturing workers (automobile assembly, seafood processing, tire manufacturing, eyeglass manufacturing, etc.) from various countries (Japan, Turkey, Korea, the United States, Portugal, France, Brazil, Italy, Denmark, and India). Intervention duration ranged from 8 weeks to 9 months. Programs included exercise-only interventions and multicomponent interventions combining exercise with non-exercise treatments. Participant characteristics also varied across studies (Table 1).

The studies of automotive workers with hand osteoarthritis [15] and metalworkers [16] included only men, whereas the study of eyewear manufacturing workers [21] included only women. Other studies included mixed-sex populations, while sex information was recorded as not reported for some studies in Table 1. Mean age was also unavailable for one study in the table. Mean age and sex distribution were summarized descriptively; formal moderator analyses were not retained in the revised synthesis. BMI, habitual physical activity, previous exercise habits, employment duration, duration of occupational exposure, baseline pain severity, comorbidities, and previous rehabilitation or treatment were not evaluated as moderators. Additional participant information was available in some reports. Nassif et al. [12] reported baseline BMI and sports participation and stated that medical history was collected. Park et al. [14] reported height, weight, and years of service. Rasotto et al. [16] reported BMI and excluded workers who had participated in structured physical activity or exercise during the preceding six months. Baseline pain scores were available in several reports used to calculate change scores. These details were not analyzed as treatment-effect modifiers. Employment duration should not be interpreted as equivalent to the duration of exposure to a specific occupational task, and medical exclusion criteria do not provide a complete description of participants’ comorbidities or previous treatment.

**Table 1:** Characteristics of Included Studies and Exercise-Based Interventions.

Study (Author & Year)	Country	Study Design	Participant Characteristics	Sample Size (IG/CG)	Mean Age (IG/CG)	Sex (IG/CG)	IG Program	CG Description
Asada et al. [18]	Japan	RCT	Manufacturing workers with or at risk of non-specific low back pain (NSLBP)	67/69	46.10 ± 11.10/45.70 ± 12.10	M 62, F 5/M 64, F 5	Hamstring stretching and spinal rotation/flexion exercises; 3 min/session, ≥once/day; 3 months total	No exercise instruction; maintained usual daily routine
Atasoy et al. [19]	Turkey	RCT	Textile factory workers with neck pain	30/30	27.20 ± 7.65/30.10 ± 4.38	M 8, F 22/M 13, F 17	Ergonomic education plus cervical exercise and manual therapy for 12 weeks. Exercise: 40–45 min/session, 3 sessions/week, including warm-up, stretching, stabilization, and low-resistance isometric exercises. Initial exercise instruction was supervised by a physiotherapist, followed by a home program with exercise tracking and weekly reminders. Manual therapy: 15 min/session, 3 sessions/week.	Individual ergonomic education, also provided to the intervention group: approximately 2 h at study entry, supported by demonstrations and written materials.
Kang et al. [15]	Korea	RCT	Automotive assembly line workers with hand osteoarthritis	15/14	46.70 ± 4.60/47.90 ± 4.00	All male (15/14)	Paraffin bath (30 min) + finger extension and strengthening exercises (30 min); 60 min/session, 5×/week; 8 weeks total	Paraffin bath (30 min) only
Lowe et al. [11]	USA	Work-group randomized trial with a repeated-measures design	Automotive manufacturing workers performing overhead assembly tasks	Initially enrolled: 41/35; completed the initial questionnaire: 37/29.	33.30 ± 8.61/37.40 ± 10.26	NR/NR	Resistance-band strengthening and pectoralis/trapezius stretching; sessions offered during a 15-min period before the work shift, next to the assembly line, for 6 months. Participation was voluntary and supervised by certified athletic trainers. Participation time was compensated at the regular hourly wage. Overall attendance was 35.1% of available sessions, corresponding to approximately 1.8 sessions/week.	No intervention; usual work activities
Moreira-Silva et al. [17]	Portugal	RCT	Workers at a multinational manufacturing plant involving repetitive and physically demanding tasks	39/31	38.00 ± 6.90/38.80 ± 8.60	NR/NR	Full-body stretching and muscular strengthening exercises performed during work hours; 10–15 min/session, 3×/week; 6 months total	Maintained usual daily activities
Nassif et al. [12]	France	RCT	Automotive assembly line workers with chronic low back pain	Randomized: 37/38; analyzed at 2 months: 32/28; analyzed at 6 months: 29/23	Baseline: 45.13 ± 9.11/45.34 ± 8.80	Baseline: M 26, F 11/M 17, F 21	Supervised exercise (45 min: warm-up, proprioceptive and coordination exercises, strengthening/endurance, and stretching) followed by hot-pack treatment (15 min); 60 min/session, 3 sessions/week for 2 months, delivered in the workplace medical department. Exercise intensity was individually adapted.	No direct study intervention; external consultations were permitted. Both groups received advice on physical activity and appropriate working postures as part of workplace policy.
Park et al. [14]	Korea	RCT	Tire factory workers with chronic low back pain	‡ LSE: 8, NWE: 8/8	LSE: 43.37 ± 5.42, NWE: 44.12 ± 5.48/45.50 ± 5.34	NR/NR	Conventional physical therapy (hot pack, interferential current therapy, and ultrasound) plus lumbar stabilization exercise (LSE) or Nintendo Wii-based exercise (NWE); 3 sessions/week for 8 weeks.	The same conventional physical therapy (hot pack, interferential current therapy, and ultrasound), without additional exercise.
Pehlevan & Şevgin [13]	Turkey	RCT	Production workers at a plastic molding factory with chronic low back pain	27/27	NR/NR	M 23, F 4/M 19, F 8	Ergonomic education plus stretching and core stabilization exercises; approximately 20–30 min/session, 3 sessions/week for 8 weeks. Initial exercises were performed with a physiotherapist, followed by an individual home program supported by written instructions, an exercise tracking chart, and weekly reminder messages. Exercise movements were adapted to participant capacity.	Individual ergonomic education, also provided to the intervention group: approximately 2 h at study entry, using visual materials, practical demonstrations, and an information booklet.
Pereira et al. [20]	Brazil	RCT	Workers in the sewing and finishing departments of a garment factory	44/17	28.70 ± 8.80/27.80 ± 7.40	M 25.0%, F 75.0%/M 41.2%, F 58.8%	Workplace physical activity program comprising stretching (40%), muscular endurance exercises (40%), relaxation, massage and self-massage techniques (10%), and group dynamics (10%).	Work stoppage and relocation to a rest area; no specific intervention
Rasotto et al. [21]	Italy	RCT	Female workers in the eyewear manufacturing industry performing manual precision assembly and finishing tasks	30/30	38.05 ± 6.07/40.32 ± 6.32	F 30/F 30	Customized upper extremity exercises (stretching, active ROM, strengthening); 30 min/session, 2×/week; 6 months total	Maintained usual daily activities; no specific intervention
Rasotto et al. [16]	Italy	RCT	Male metalworking assembly line workers at high risk for upper extremity MSDs	17/34	41.65 ± 8.26/40.88 ± 7.55	M 17/M 34	Tailored upper-extremity exercises, including active mobilization, strengthening, and stretching; approximately 30 min/session, twice weekly; 9 months of exercise delivery within a 10-month study period, accounting for the planned summer closure. Sessions were supervised by an exercise specialist. Strengthening intensity was maintained at 5–7/10 and stretching intensity at 5–6/10. Outcomes were assessed at baseline, 5 months, and 10 months.	Maintained usual daily activities; no specific intervention
Sundstrup et al. [22] †	Denmark	RCT	Slaughterhouse workers with chronic upper extremity pain and work disability	33/33	48.00 ± 9.00/43.00 ± 9.00	M 25, F 8/M 26, F 7	Supervised resistance exercises targeting the shoulder, arm, and hand muscles; 10 min/session, 3 sessions/week for 10 weeks. Training loads progressed from 20 to 8 repetition maximum (RM). Sessions were conducted in designated workplace training rooms, with home-training equipment provided for absences from work. Reported exercise adherence was 81% (mean 2.4 of 3 prescribed sessions/week).	Individual ergonomic instruction and training (usual care)
Sundstrup et al. [23] †	Denmark	RCT	Slaughterhouse workers with chronic upper extremity pain and work disability	27/31 (per fatigue analysis)	48.00 ± 9.00/43.00 ± 9.00	M 25, F 8/M 26, F 7 (initial allocation)	Supervised resistance exercises targeting the shoulder, arm, and hand muscles; 10 min/session, 3 sessions/week for 10 weeks. Training loads progressed from 20 to 8 repetition maximum (RM). Sessions were conducted in designated workplace training rooms, with home-training equipment provided for absences from work. Reported exercise adherence was 81% (mean 2.4 of 3 prescribed sessions/week).	General ergonomic instruction (usual care)
Gundmi et al. [10]	India	Cluster-RCT	Seafood processing workers reporting upper extremity MSDs	91/93	39.65 ± 10.29/42.31 ± 10.24	M 41, F 50/M 19, F 74	SEBI (stretching, strengthening, active ROM exercises) with education; 30 min/session, 3–5×/week; 12 weeks total	Maintained usual daily work activities; no specific intervention

RCT, Randomized Controlled Trial; CCT, Controlled Clinical Trial; Cluster-RCT, Cluster Randomized Controlled Trial; IG, Intervention Group; CG, Control Group; M, Male; F, Female; NR, Not Reported; NSLBP, Non-Specific Low Back Pain; LSE, Lumbar Stabilization Exercise; NWE, Nintendo Wii-based Exercise; ROM, Range of Motion; RM, Repetition Maximum; MSD, Musculoskeletal Disorder; SEBI, Stretching and Exercise-Based Intervention; JOEM, Journal of Occupational and Environmental Medicine; MT, Manual Therapy. † Sundstrup et al. [22,23] are reports from the same randomized trial of 66 participants and were counted as one study. Their pain and work-ability findings were retained in the narrative synthesis and were not included in the revised primary pooled analyses. ‡ Park et al. [14] randomized 24 participants to lumbar stabilization exercise, Nintendo Wii-based exercise, or a shared control group (*n* = 8 each). In the revised analyses, the two exercise groups were combined into one intervention group (*n* = 16), and the shared control group (*n* = 8) was included once per outcome. All three groups received conventional physical therapy.

### 3.2. Risk of Bias Results

The review included 11 individually randomized trials, represented by 12 reports, and two trials with group-level randomization: Lowe et al. [11] and Gundmi et al. [10]. The assessments presented in Supplementary S2 and S3 concern the individually randomized trial reports; the two Sundstrup reports describe the same underlying trial. Supplementary S2 incorporates the revised judgments for the two-month pain and disability outcomes in Nassif et al.; the judgments for the other reports were retained from the original assessment. Supplementary S3 summarizes the 12 reports with equal report weights. These percentages describe reports rather than independent trials and do not constitute separate risk-of-bias summaries for each revised outcome domain. Supplementary S4 presents the cluster-specific assessment for Gundmi et al. [10]. Lowe et al. [11] reported randomization at the work-group level, with allocation of the exercise opportunity determined by a coin flip. The study is therefore described as a work-group randomized trial rather than a non-randomized controlled trial.

In the reported assessments of individually randomized trials, the randomization-process domain was rated as low risk for most reports, whereas Pereira et al. [20] was rated as high risk. Most reports were rated as having some concerns in the domain of bias due to deviations from intended interventions (Supplementary S2). For Gundmi et al. [10], the reported cluster-specific assessment indicated some concerns regarding the randomization process, the timing of participant identification and recruitment, and deviations from intended interventions. Bias due to missing outcome data was rated as high risk, resulting in an overall high-risk judgment (Supplementary S4). These domain-level judgments should be distinguished from the separate question of whether the effect estimate used in a meta-analysis adequately accounts for clustering. The inability to blind participants and intervention providers does not, by itself, establish a high risk of bias. For bias due to deviations from intended interventions, the relevant considerations include whether awareness of allocation led to deviations arising from the trial context that could affect the outcome and whether the analysis was appropriate for the effect of interest. For bias in measurement of the outcome, awareness of allocation and its potential influence on outcome assessment require separate consideration. The use of standardized instruments such as VAS or NRS does not, by itself, establish a low risk of measurement bias. For the two-month pain and disability outcomes in Nassif et al. [12], re-examination of the report identified concerns regarding allocation concealment, changes in physical activity outside the assigned intervention, missing outcome data, and the absence of an available prespecified analysis plan. At two months, 32 of 37 intervention participants and 28 of 38 control participants were analyzed. The report did not establish that the missing outcomes were unrelated to participants’ underlying pain or disability. Outcome measurement was judged to be at high risk of bias because the NRS and RMDQ were self-reported by participants who knew their allocation, and knowledge of allocation was considered likely to influence these subjective reports. The overall judgment was therefore high risk for these outcomes.

For Lowe et al. [11], the principal methodological concerns were recruitment with knowledge of work-group allocation, alignment of the exercise condition with work shift, incomplete longitudinal questionnaire responses, and uncertainty about accounting for work-group clustering. Participants knew their group allocation at enrollment, and the recruitment information differed between the exercise and control groups. The reported mixed models included random effects for worker and time, but did not explicitly describe a work-group random effect. These features warrant caution when interpreting the study’s findings. The previous ROBINS-I classification does not correspond to the reported randomized design and is not retained as the revised risk-of-bias judgment.

### 3.3. Meta-Analyses

#### Meta-Analyses on Pain and Function

**Figure 2:**
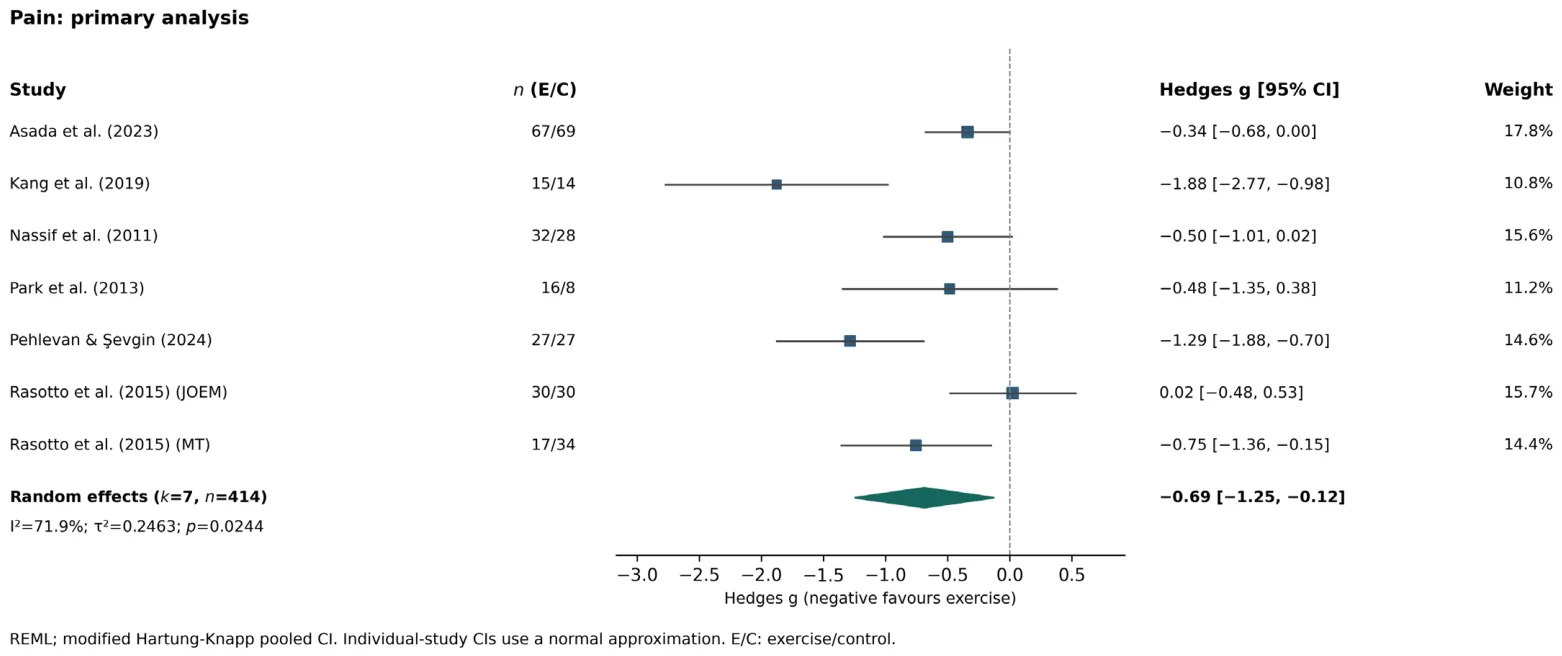
Forest plot of exercise-based interventions for pain in the revised primary synthesis. Studies included in this analysis are reported in references [12,13,14,15,16,18,21]. Effects are expressed as Hedges’ g, with negative values favouring exercise. Squares represent individual-study estimates, with areas proportional to random-effects weights; horizontal lines indicate 95% confidence intervals. The diamond represents the pooled estimate and its modified Hartung–Knapp 95% confidence interval, with between-study variance estimated using REML. For Park et al. [14], both exercise arms were combined, and the shared control group was included once. E/C denotes exercise/control sample sizes. JOEM and MT distinguish the two Rasotto reports published in 2015. The vertical dashed line indicates no effect (Hedges’ g = 0).

Seven studies involving 414 participants contributed to the revised primary pain analysis [12,13,14,15,16,18,21]. The pooled effect favoured exercise-based interventions over the included control conditions (SMD = −0.69, 95% CI: −1.25 to −0.12; *p* = 0.0244). Between-study heterogeneity was substantial (I^2^ = 71.9%; τ^2^ = 0.2463; Cochran’s Q test, *p* = 0.0016). This estimate represents an average across different musculoskeletal conditions, occupational settings, and treatment comparisons rather than a uniform effect expected in all manufacturing workers. Individual-study estimates and the pooled pain effect are presented in Figure 2.

The revised primary synthesis excluded the multidimensional NBQ total score, approximate data derived from medians and interquartile ranges, and studies for which compatible change-score data or an appropriate variance could not be established. Studies excluded from this quantitative synthesis remained part of the review and were considered descriptively or in sensitivity analyses where feasible.

Functional outcomes were synthesized separately by clinical domain rather than combined into a single overall physical-function estimate. Three studies involving 143 participants contributed to the disability and activity-limitation synthesis, using the AUSCAN physical-function subscale, the Roland–Morris Disability Questionnaire, and the Oswestry Disability Index [12,13,15]. The pooled estimate favoured exercise, but its confidence interval included no effect (SMD = −0.67, 95% CI: −1.42 to 0.07; *p* = 0.0599; I^2^ = 0.0%; τ^2^ = 0). The absence of detected statistical heterogeneity does not establish clinical equivalence between these instruments or populations.

**Figure 3:**
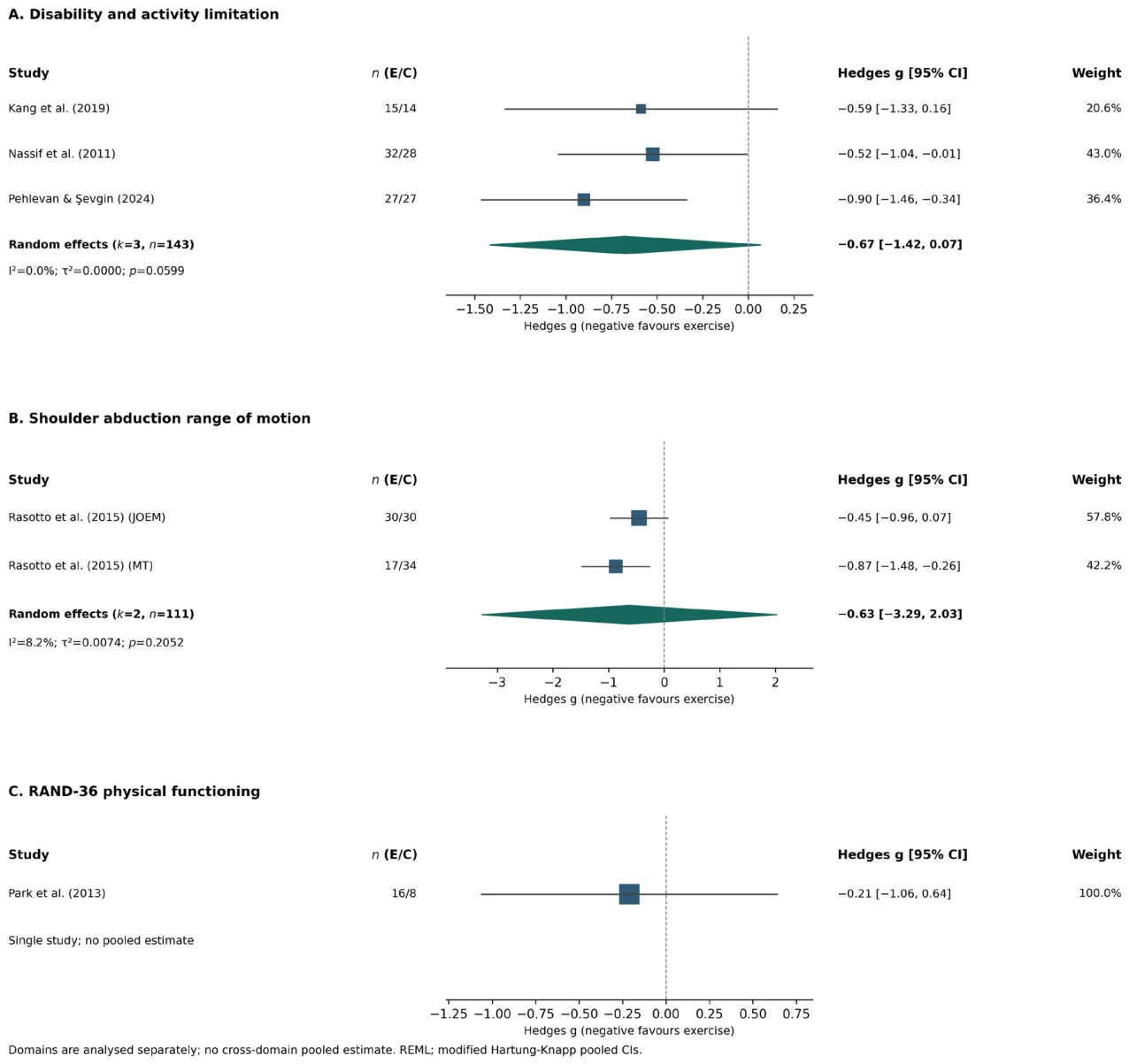
Forest plots of functional outcomes analysed separately by domain: (**A**) disability and activity limitation, (**B**) shoulder abduction range of motion, and (**C**) RAND-36 physical functioning. Studies included in these analyses are reported in references [12,13,14,15,16,21]. No overall effect was pooled across these domains. Effects are expressed as Hedges’ g, with negative values favouring exercise. Squares represent individual-study estimates, with areas proportional to the weights within each panel; horizontal lines indicate 95% confidence intervals. Diamonds in panels A and B represent the pooled estimates and their 95% confidence intervals. The vertical dashed line indicates no effect (Hedges’ g = 0). Individual-study confidence intervals use a normal approximation; pooled confidence intervals use the modified Hartung–Knapp method with REML estimation of between-study variance. Panel C presents a single-study estimate from Park et al. [14], with both exercise arms combined and the shared control group included once. Weights are calculated within each panel. E/C denotes exercise/control sample sizes.

Shoulder abduction range of motion was analysed separately in two studies involving 111 participants [16,21]. The pooled estimate was imprecise (SMD = −0.63, 95% CI: −3.29 to 2.03; *p* = 0.2052; I^2^ = 8.2%). For RAND-36 physical functioning, the combined exercise arms in Park et al. [14] contributed one comparison involving 24 participants (SMD = −0.21, 95% CI: −1.06 to 0.64). This single-study estimate was not pooled with disability or range-of-motion measures. These findings do not establish a consistent benefit across functional domains. The domain-specific results are presented in Figure 3A–C.

### 3.4. Intervention Composition

Intervention composition was summarized descriptively in Table 1. Formal comparisons between single-component and multicomponent programs were not retained in the revised quantitative synthesis because few studies were available within the revised outcome domains. The descriptive classification does not establish equivalence, interchangeability, or superiority between program types, nor does it isolate the effects of individual exercise or non-exercise components.

### 3.5. Meta-Regression

Formal meta-regression was not retained in the revised quantitative synthesis because few studies were available within each revised outcome domain. The regression coefficients and *p*-values from the original datasets are therefore not presented as results of the revised analyses. The revised synthesis does not establish associations between intervention effectiveness and intervention duration, weekly exercise volume, mean age, or female proportion.

### 3.6. Publication Bias

Following revision of the quantitative datasets, formal funnel-plot asymmetry testing and trim-and-fill analyses were not retained because few studies were available within each revised outcome domain. With limited numbers of studies, funnel-plot asymmetry tests have low power, and a nonsignificant result would not demonstrate the absence of publication bias. Moreover, differences in clinical characteristics and study methods may contribute to funnel-plot asymmetry, making it difficult to attribute any observed asymmetry specifically to publication bias.

Trim-and-fill also does not provide a definitive test for publication bias or establish an unbiased treatment effect. Studies imputed by this method are hypothetical rather than identified unpublished studies, and the adjusted estimate depends on the method’s assumptions. An adjusted confidence interval excluding the null would therefore not establish that publication bias was absent or adequately addressed. Accordingly, the estimates previously calculated from the original datasets are not presented as results of the revised synthesis. Publication bias could not be excluded for either pain or the revised functional outcome domains, and its potential influence on the pooled estimates remains uncertain.

### 3.7. Sensitivity Analysis

Sensitivity analyses examined the influence of imputed change-score standard deviations and uncertain source data. For pain, assuming baseline–follow-up correlations of 0.25 and 0.75 yielded pooled SMDs of −0.64 (95% CI: −1.21 to −0.06) and −0.80 (95% CI: −1.35 to −0.25), respectively. Substituting the change-score statistics reported in Table 4 of Rasotto et al. [16] yielded an SMD of −0.67 (95% CI: −1.23 to −0.11). Excluding that study yielded an SMD of −0.69 (95% CI: −1.39 to 0.02). Across leave-one-out pain analyses, point estimates ranged from −0.81 to −0.53, but not all confidence intervals excluded no effect. Thus, the direction of the pooled estimate was consistent, whereas statistical significance depended on the included studies.

Including the approximate data from Moreira-Silva et al. [17] yielded a pooled SMD of −0.68 (95% CI −1.14 to −0.22; eight studies, 484 participants; I^2^ = 67.7%). This analysis was supplementary because the study input relied on approximations from reported medians and interquartile ranges; it did not replace the primary synthesis.

For disability and activity limitation, correlations of 0.25 and 0.75 yielded SMDs of −0.63 (95% CI: −1.37 to 0.11) and −0.77 (95% CI: −1.52 to −0.02), respectively. All leave-one-out confidence intervals included no effect. These findings indicate uncertainty related to the small number of studies and the assumptions used to estimate change-score variability. The leave-one-out results for pain and disability/activity limitation are presented in Supplementary S5 and Supplementary S6, respectively.

The range-of-motion synthesis was particularly sensitive to the discrepant statistics in Rasotto et al. [16]. Using its Table 4 change scores yielded an SMD of −1.16 (95% CI: −10.50 to 8.17; I^2^ = 90.9%). Neither version provided a precise pooled estimate, and the unresolved source-data discrepancy further limits interpretation.

For shoulder abduction range of motion, assuming baseline–follow-up correlations of 0.25 and 0.75 yielded pooled SMDs of −0.53 (95% CI −3.05 to 1.99) and −0.81 (95% CI −3.98 to 2.36), respectively. For the single-study RAND-36 physical-functioning comparison, the corresponding estimates were −0.17 (95% CI −1.02 to 0.68) and −0.29 (95% CI −1.15 to 0.56). All of these confidence intervals included no effect.

### 3.8. Certainty of Evidence

The GRADE assessment indicated very low certainty for pain intensity, low certainty for disability/function-status outcomes, and very low certainty for shoulder abduction range of motion and RAND-36 physical functioning (Supplementary S7). For pain intensity, the certainty of evidence was very low, reflecting one-level downgrades for risk of bias, inconsistency, and imprecision. The revised synthesis included seven studies with 414 participants and showed substantial heterogeneity (I^2^ = 71.9%). The pooled SMD was −0.69 (95% CI −1.25 to −0.12); although the confidence interval excluded no effect, it encompassed markedly different magnitudes of benefit, limiting confidence in the size of the effect. For disability/function-status outcomes, certainty was low after one-level downgrades for risk of bias and imprecision. The synthesis included three studies with 143 participants (SMD −0.67, 95% CI −1.42 to 0.07). For shoulder abduction range of motion, certainty was very low after a one-level downgrade for risk of bias and a two-level downgrade for imprecision; two studies with 111 participants contributed data (SMD −0.63, 95% CI −3.29 to 2.03). For RAND-36 physical functioning, evidence from one trial with 24 participants was rated as very low certainty because of serious risk of bias and very serious imprecision (SMD −0.21, 95% CI −1.06 to 0.64). These assessments apply to the specified outcome domains rather than to physical function as a single construct. Publication bias could not be excluded, and limitations of publication-bias testing were not used as reasons for downgrading imprecision.

## 4. Discussion

This study systematically examined the impact of exercise-based interventions on work-related musculoskeletal disorders (WMSDs) in manufacturing workers. The included populations encompassed workers with existing musculoskeletal symptoms or conditions and workers described as being at risk. Because the pooled outcomes were pain intensity and physical function rather than the incidence of new disorders, the findings primarily inform symptom management and functional improvement rather than primary prevention. The revised primary pain synthesis favored exercise-based interventions (SMD = −0.69, 95% CI −1.25 to −0.12), with substantial heterogeneity (I^2^ = 71.9%). Functional outcomes were analyzed separately by domain, and their confidence intervals included no effect. The pooled pain estimate represents an average across clinically diverse studies rather than a uniform benefit. Differences in anatomical region, baseline pain severity, exercise type, intervention duration, co-interventions, outcome instruments, and occupational exposure are plausible contributors to this variability. Baseline symptom severity may affect the scope for improvement, while differences in pain instruments, recall periods, and the body regions assessed may affect the meaning and comparability of the measured changes. Exercise prescriptions and accompanying treatments may also produce different effects under different occupational demands. Methodological differences, including study design, handling of missing outcome data, outcome assessment procedures, and the analysis of clustered or repeated observations, may also contribute to differences in effect estimates or their precision. These explanations remain hypotheses because the revised analyses did not establish their respective contributions. Substantial heterogeneity and the sensitivity of statistical significance to study inclusion limit confidence in applying the pooled estimate to a particular occupational or clinical setting. Clinically, the pooled SMD should not be treated as the expected benefit for every manufacturing worker or used alone to select an exercise program. Its applicability requires consideration of the worker’s symptoms, anatomical region, occupational demands, and the intervention and comparator represented in the relevant studies. The mechanisms underlying the observed changes in pain and physical function cannot be determined from the present meta-analysis. Potential explanations involving changes in muscle endurance, local blood flow, or pain modulation should be regarded as hypotheses rather than mechanisms established by the pooled findings. The results therefore support cautious interpretation of the measured clinical outcomes without attributing them to a specific physiological pathway. Previous reviews have examined exercise interventions in manual workers [5] and workplace interventions in employees with physically demanding work [6], reporting benefits of exercise for musculoskeletal outcomes. The present review complements this literature by focusing specifically on manufacturing workers and synthesizing controlled studies identified through 31 March 2026. Its contribution is to provide a pooled estimate for pain and domain-specific estimates for functional outcomes, alongside an assessment of evidence certainty and the limitations affecting their application within manufacturing settings. This occupational focus does not eliminate differences between jobs, clinical conditions, or interventions. Accordingly, the findings should be used to distinguish what the available evidence suggests from what remains unresolved: which workers and conditions benefit most, which exercise prescriptions are appropriate, and how workplace demands and comparator treatments influence the observed effects.

Occupational and task-related heterogeneity should be considered when interpreting these findings. The included studies encompassed automobile assembly, textile and garment production, tire and plastic manufacturing, eyewear and metalworking assembly, slaughterhouse work, and seafood processing (Table 1). The task descriptions included overhead assembly [11], repetitive and physically demanding work [17], and manual precision assembly and finishing [21]. These tasks involve different patterns of mechanical loading and may differ in movement repetition, force requirements, working posture, and opportunities for recovery. Such differences could influence musculoskeletal symptoms and responses to exercise and may contribute to the variability in pain outcomes; however, their contribution was not directly examined in this review. Accordingly, the pooled estimates summarize effects across diverse occupational settings and should not be interpreted as demonstrating a uniform benefit across all manufacturing tasks. Application of these findings to a particular workplace requires consideration of its specific task demands and the musculoskeletal conditions being addressed. The present review cannot determine which occupational groups are most likely to benefit.

Anatomical and clinical heterogeneity also limits the interpretation of the pooled findings. The included populations comprised workers with nonspecific or chronic low back pain, neck pain, hand osteoarthritis, and other upper-extremity musculoskeletal disorders (Table 1). These conditions differ in their underlying pathology, clinical course, affected activities, and exercise requirements. Consequently, a pooled effect for pain does not establish that exercise produces comparable benefits across these conditions. Measurement constructs differed across the eligible studies. Because the NBQ total score incorporates dimensions beyond pain intensity, it was excluded from the revised primary pain synthesis. This exclusion improved the specificity of the pain outcome, although differences between pain instruments, anatomical regions, and clinical populations remained. Interpretation of the physical-function estimate also depends on what each instrument measures. Range of motion and muscle strength assess aspects of physical impairment or capacity, whereas disability and functional-status questionnaires assess limitations in activities or perceived functioning. These outcomes may be related, but they are not interchangeable. Even when effect directions are aligned, expressing results as SMDs does not resolve differences in the underlying constructs. An overall estimate should therefore not be interpreted as demonstrating comparable improvements in joint mobility, strength, and everyday functioning. In the revised synthesis, functional outcomes were analyzed separately as disability/activity limitation, shoulder abduction range of motion, and RAND-36 physical functioning. This separation improved conceptual clarity, but the small number of studies and differences within domains limit the specificity of clinical conclusions. The domain-specific estimates should not be interpreted as establishing condition-specific benefits. Whether the magnitude of benefit differs between anatomical regions remains unresolved and requires appropriately defined subgroup comparisons.

Participant characteristics beyond age and sex may also influence the interpretation and applicability of these findings. BMI, habitual physical activity, and previous exercise habits may be associated with physical capacity and tolerance of exercise. Employment duration and years of exposure to specific tasks may reflect cumulative occupational loading, while baseline pain severity may influence the scope for improvement. Comorbidities and previous rehabilitation or treatment may also affect baseline function, exercise participation, and treatment response. These variables were not evaluated as moderators in the present review, and their contribution to differences between studies remains uncertain. Formal moderator analyses were not retained in the revised synthesis; therefore, the review does not establish associations between treatment effects and age or sex. More generally, study-level demographic summaries cannot establish individual-level differences in treatment response.

Both single-component and multicomponent programs were represented in the review. Formal comparisons between these categories were not retained in the revised quantitative synthesis. The descriptive classification does not establish equivalence, interchangeability, or superiority between the approaches. This classification describes the composition of the full intervention program, rather than the number of exercise modalities or the treatment difference between groups. Consequently, a multicomponent program may evaluate either the addition of exercise to a shared background treatment or the combined effect of exercise and other components delivered only to the intervention group. This descriptive classification does not isolate the contribution of individual components or establish whether adding a particular non-exercise intervention improves outcomes.

The included programs encompassed stretching, strengthening, core stabilization, high-intensity resistance exercise, range-of-motion exercise, and Wii-based exercise, sometimes combined with non-exercise interventions (Table 1). This diversity limits conclusions about which exercise prescription is most appropriate for a particular occupational task or musculoskeletal condition. The revised synthesis did not evaluate intervention duration, weekly exercise volume, or exercise intensity through formal moderator analyses. Duration and weekly exercise time alone do not adequately characterize exercise intensity, and the available analyses do not establish an optimal dose. Moreover, benefits observed with combined interventions cannot necessarily be attributed to exercise alone. Future trials should clearly specify exercise intensity and progression, distinguish actual exercise time from total treatment time, and evaluate prescriptions tailored to defined occupational demands and musculoskeletal conditions.

The included studies provide examples of exercise approaches investigated in specific occupational and clinical contexts. For workers with chronic low back pain, interventions included lumbar stabilization or Nintendo Wii-based exercise in tire factory workers [14] and stretching with core stabilization in plastic manufacturing workers [13]. For upper-extremity conditions, programs included finger exercises combined with paraffin therapy in automotive workers with hand osteoarthritis [15], customized stretching, active range-of-motion, and strengthening exercises in eyewear manufacturing workers [21], and high-intensity resistance exercise in slaughterhouse workers with chronic upper-extremity pain [22,23]. These examples describe the interventions studied rather than establish a preferred prescription. In Park et al. [14], both exercise arms were combined for the revised analyses, estimating the average effect of adding either exercise approach to conventional physical therapy. This combined comparison does not establish which exercise approach is more effective, and benefits from programs containing additional treatments cannot necessarily be attributed to exercise alone. The review did not establish comparative effectiveness by exercise type or evaluate the prevention of new WMSDs. Accordingly, these programs should be considered examples for further evaluation in defined worker populations, rather than exercise-specific prevention recommendations.

Heterogeneity in control conditions and co-interventions also affects the interpretation of the findings. Comparators included no intervention or usual work activities, ergonomic education, and active treatments such as paraffin therapy and conventional physical therapy (Table 1). Where the same non-exercise treatment was provided to both groups, the comparison estimates the additional effect of exercise in that treatment context. Examples include exercise plus paraffin therapy versus paraffin therapy alone [15], and exercise added to conventional physical therapy versus the same physical therapy without additional exercise [14]. In contrast, exercise combined with manual therapy and ergonomic education versus ergonomic education alone [19] estimates the combined additional effect of exercise and manual therapy. Similarly, the program evaluated by Pereira et al. [20] included stretching and muscular endurance exercises together with relaxation, massage, self-massage, and group dynamics; its effect cannot be attributed exclusively to exercise. The pooled estimates therefore summarize different treatment comparisons and should not be interpreted as a uniform estimate of the effect of exercise alone.

The available evidence was insufficient to establish whether intervention duration, weekly exercise volume, mean age, or female proportion modifies intervention effectiveness. Formal meta-regression was not retained in the revised synthesis, and no dose–response relationship or demographic treatment recommendation can be inferred. Future adequately powered trials should directly compare exercise prescriptions in clearly characterized worker populations. Including more studies could improve the precision of future analyses, but would not necessarily produce larger treatment effects or resolve clinical heterogeneity.

The certainty of evidence differed across outcome domains: very low for pain, low for disability/function-status outcomes, and very low for shoulder abduction range of motion and RAND-36 physical functioning. Exercise-based interventions may improve disability/function-status outcomes, but the confidence interval also included little or no difference. Evidence concerning pain, shoulder range of motion, and RAND-36 physical functioning remains very uncertain. These findings do not establish a consistent improvement across all aspects of physical function. Although the leave-one-out pain estimates consistently favored exercise in direction, some confidence intervals included no effect. This sensitivity to study inclusion, together with the limited certainty of evidence, warrants caution; statistical significance alone does not establish clinical importance. Publication bias could not be excluded. The small number of studies within the revised outcome domains limited the informativeness of formal funnel-plot asymmetry testing and trim-and-fill, and these analyses were not retained. Together with the substantial heterogeneity in pain outcomes, these findings warrant caution when applying the pooled estimates to specific manufacturing settings. Moreover, cost-effectiveness was not evaluated in this review, and no conclusion about economic value can be drawn. Further rigorously designed, adequately powered trials and separate economic evaluations are needed.

For occupational rehabilitation, the practical contribution of this review is to clarify the limits of applying an overall exercise effect to an individual worker or workplace. The findings do not establish which occupational or clinical subgroups benefit most or support a preferred exercise type, intensity, frequency, or duration. Application therefore requires consideration of whether the worker’s condition, task demands, and available treatment options resemble those represented in the included studies. To address these gaps, future trials should enroll clearly characterized occupational and clinical populations, compare well-defined exercise prescriptions against clearly specified controls, and report adherence, adverse events, supervision, delivery during working hours, and sustained outcomes. Such information is needed to determine both the effectiveness and practical suitability of programs for particular manufacturing settings.

The search was limited to five bibliographic databases, without dedicated searches of grey literature sources, trial registries, dissertation repositories, or conference proceedings. Together with the restriction to peer-reviewed publications, this may have resulted in the omission of unpublished or otherwise difficult-to-locate evidence. Consequently, publication bias cannot be excluded, and the pooled estimates may overstate benefits if studies with unfavorable or nonsignificant findings were less likely to be published or retrieved.

This review has several limitations. First, the included studies differed in occupational setting, task demands, exercise protocols, control conditions, anatomical regions, and follow-up periods. These differences may have contributed to the substantial heterogeneity in pain outcomes (I^2^ = 71.9%). Furthermore, pooling outcomes across anatomical regions and clinical conditions may have obscured clinically meaningful differences in intervention effects. Separating functional outcomes into disability/activity limitation, shoulder range of motion, and RAND-36 physical functioning improved conceptual clarity, but did not eliminate differences between instruments and populations within domains. The small number of studies and imprecise estimates limit domain-specific conclusions. In addition, assumptions used to estimate change-score variability and unresolved discrepancies in some source statistics introduce uncertainty, as examined in the sensitivity analyses. Because subgroup analyses by anatomical region were not performed, the present review cannot establish whether the observed benefits apply similarly to workers with low back pain, neck pain, hand osteoarthritis, and other upper-extremity disorders. Subgroup analyses by occupation or predominant physical exposure were not undertaken because the studies were distributed across multiple occupational settings, many represented by a single independent study, limiting the reliability of comparisons between occupational subgroups. Moreover, grouping studies solely by industry would not reliably distinguish predominant task-level exposures. Consequently, this review could not establish whether intervention effects differed according to occupational task or physical exposure, which limits occupation-specific recommendations. Future trials should report task characteristics and the intensity and duration of occupational exposures using standardized measures to support more informative comparisons. The influence of control conditions was not separately evaluated through comparator-based subgroup or sensitivity analyses. Consequently, this review cannot determine whether the magnitude of the pooled effects differed between studies using no intervention or usual work, ergonomic education, or active treatment comparators. The inclusion of programs combining exercise with non-exercise components also limits attribution of the pooled effects to exercise alone. Classification by overall intervention composition does not resolve differences between shared background treatments and additional components delivered only to the intervention group. The separate contributions of individual components therefore remain uncertain. Participant-related sources of heterogeneity, including age and sex distribution, were not evaluated through formal moderator analyses in the revised synthesis. Although some reports provided additional participant characteristics, these data were not assembled into a complete, harmonized study-level dataset for moderator analysis. Their potential influence on intervention effects was therefore not estimated, limiting the identification of participant groups most likely to benefit. Intervention adherence was not evaluated as a potential source of variation in intervention effects. Consequently, the review cannot determine whether differences in the exercise actually completed, rather than the prescribed exercise dose alone, contributed to differences between studies. The present review also does not provide a systematic synthesis of exercise-related adverse events. Therefore, its findings on pain and physical function should not be interpreted as establishing the safety of the included exercise programs.

In the three-arm trial by Park et al. [14], the two exercise arms were combined to incorporate both eligible exercise approaches while including the shared control group only once. However, combining lumbar stabilization and Nintendo Wii exercise obscures differences between these approaches. The combined estimate therefore does not identify the more effective exercise modality.

Lowe et al. [11] and Gundmi et al. [10] were retained in the review but did not contribute to the revised primary pooled estimates because compatible effect estimates with adequately documented variances accounting for their group-level allocation could not be established from the available materials. This limits the coverage of the quantitative synthesis. Assessment of cluster-specific risk of bias and statistical adjustment for clustering are distinct requirements; the presence of a cluster-specific risk-of-bias assessment does not establish that an effect estimate adequately accounts for clustering.

Second, the limited number of studies, particularly for physical function and moderator analyses, restricts the precision of the evidence and the ability to investigate differences between studies. Additional eligible studies could improve precision and statistical power, but would not necessarily produce larger or statistically significant effects. The contribution of further evidence would also depend on study quality, sample size, and clinical comparability; additional studies could strengthen, attenuate, or change the current estimates.

Thirdly, the long-term sustainability of intervention effects is challenging to evaluate due to the limited duration of follow-up periods in most studies. The minimum intervention duration of two weeks also restricted the scope of the review. Studies evaluating shorter interventions or immediate responses to exercise were outside the eligibility criteria, so the findings cannot be generalized to these interventions. Whether including such studies would change the pooled estimates remains unknown. Fourthly, given that the included studies contain data from a multinational environment and consist primarily of RCTs, there may be limitations in directly generalizing the findings to manufacturing sites in specific countries.

## 5. Conclusions

The revised primary pain synthesis favored exercise-based interventions among manufacturing workers, but the evidence for pain reduction remains very uncertain. Exercise-based interventions may improve disability/function-status outcomes based on low-certainty evidence, although the confidence interval included little or no difference. Evidence for shoulder abduction range of motion and RAND-36 physical functioning was very uncertain and did not establish a clear benefit. These findings concern symptom management and functional outcomes and do not establish the effectiveness of exercise for preventing new WMSDs. Substantial heterogeneity in pain outcomes and the limited evidence within each functional domain restrict the generalizability of the findings. The optimal exercise type, dose, intensity, intervention duration, and combination with other workplace interventions remain uncertain. The available evidence does not establish equivalence between single-component and multicomponent programs or justify selecting these approaches interchangeably. Cost-effectiveness was not evaluated. Further rigorously designed, adequately powered trials are needed to determine which exercise prescriptions and combinations are appropriate for specific occupational tasks and musculoskeletal conditions and whether benefits are sustained. Separate economic evaluations are required to assess cost-effectiveness.

## Data Availability

This systematic review and meta-analysis used aggregate data extracted from previously published study reports, which are identified in the reference list. No individual participant data were collected.

## Supplementary Materials

The following supporting information can be downloaded at https://www.mdpi.com/article/10.3390/healthcare14193230/s1, Supplementary S1: Database Search Strategies for Systematic Review; Supplementary S2: ROB2.0 traffic light plot; Supplementary S3: ROB 2.0 summary; Supplementary S4: ROB2-Cluster traffic light; Supplementary S5: Leave-One-Out Sensitivity Analysis for the Effect of Exercise Interventions on Pain Outcomes; Supplementary S6: Leave-One-Out Sensitivity Analysis for Disability and Activity Limitation; Supplementary S7: Summary of findings: Exercise-based interventions compared to control for manufacturing workers with work-related musculoskeletal disorders; Supplementary S8: PRISMA 2020 Checklist [24]. References [10,12,13,14,15,16,17,18,19,20,21,22,23] are cited in Supplementary Materials S2–S7.

## Abbreviations

The following abbreviations are used in this manuscript:

CCT	Controlled Clinical Trial
CI	Confidence Interval
GRADE	Grading of Recommendations Assessment, Development and Evaluation
NRS	Numeric Rating Scale
PRISMA	Preferred Reporting Items for Systematic Reviews and Meta-Analyses
PROSPERO	International Prospective Register of Systematic Reviews
RCT	Randomized Controlled Trial
ROBINS-I	Risk Of Bias In Non-randomized Studies of Interventions
RoB 2	Risk of Bias 2
ROM	Range of Motion
SMD	Standardized Mean Difference
VAS	Visual Analog Scale
WMSDs	Work-Related Musculoskeletal Disorders
