# Supplementary material for: Effects of Exercise-Based Interventions on Musculoskeletal Pain and Physical Function in Manufacturing Workers: A Systematic Review and Meta-Analysis
Source: Healthcare (Basel). 2026 Oct 1;14(19):3230. doi: 10.3390/healthcare14193230 (PMC13664732; doi:10.3390/healthcare14193230)
Supplement: Supplementary file 1 [file healthcare-14-03230-s001.zip › Supplementary S1.pdf]

Supplementary S1 Database-specific search strategies

The search covered each database from inception through March 31, 2026, without language restrictions.

Database	Search Strategy
**PubMed**	("Manufacturing"[tw] OR "Factory"[tw] OR "Industry"[tw] OR "Industrial workers"[tw] OR "Manual workers"[tw] OR "Occupational groups"[MeSH]) AND ("Musculoskeletal Diseases"[MeSH] OR "Musculoskeletal Disorders"[tw] OR "Occupational diseases"[MeSH] OR "Back Pain"[tw] OR "Neck Pain"[tw] OR "Myalgia"[tw]) AND ("Exercise"[MeSH] OR "Exercise Therapy"[MeSH] OR "Stretching"[tw] OR "Physical Training"[tw] OR "Gymnastics"[tw] OR "Workplace health promotion"[tw]) AND ("Clinical Trial"[pt] OR "Controlled Clinical Trial"[pt] OR "Comparative Study"[tw] OR "Randomized"[tw])
**Cochrane**	#1 [MeSH descriptor: [Manufacturing and Industrial Facilities] explode]<br>#2 (Manufacturing OR factory OR (industrial NEXT worker*) OR (manual NEXT worker*) OR SME):ti,ab<br>#3 #1 OR #2<br>#4 [MeSH descriptor: [Musculoskeletal Diseases] explode]<br>#5 ((musculoskeletal NEXT disorder*) OR WMSD OR "back pain"):ti,ab<br>#6 "neck pain":ti,ab<br>#7 "shoulder pain":ti,ab<br>#8 (repetitive NEXT strain):ti,ab<br>#9 myalgia:ti,ab<br>#10 #4 OR #5 OR #6 OR #7 OR #8 OR #9<br>#11 [MeSH descriptor: [Exercise] explode]<br>#12 (exercise OR stretching OR (physical NEXT training) OR (workplace NEXT program)):ti,ab<br>#13 #11 OR #12<br>#14 #3 AND #10 AND #13
**Embase**	('manufacturing worker'/exp OR 'industry'/exp OR 'industrial worker':ti,ab OR 'manual worker':ti,ab) AND ('musculoskeletal disease'/exp OR 'musculoskeletal pain':ti,ab OR 'back pain':ti,ab) AND ('exercise'/exp OR 'stretching exercise'/exp OR 'workplace intervention':ti,ab) AND ('clinical trial'/exp OR 'randomization':ti,ab OR 'controlled study':ti,ab)
**CINAHL**	(TX Manufacturing OR TX Industry OR TX "Industrial workers" OR TX "Manual workers") AND (MH "Musculoskeletal Diseases+" OR TX "Musculoskeletal Pain") AND (MH "Exercise+" OR TX Stretching OR TX "Workplace Health") AND (TX "Clinical Trial" OR TX Randomized OR TX "Control Group")
**Web of Science**	TS=(Manufacturing OR Factory OR Industry OR "Industrial workers" OR "Manual workers") AND TS=("Musculoskeletal Disorders" OR "Work-related Pain" OR "Back Pain" OR "Neck Pain") AND TS=(Exercise OR Stretching OR Training OR "Workplace Exercise") AND TS=(Randomized OR "Clinical Trial" OR "Controlled Trial")
