# Supplementary material for: Effects of Exercise-Based Interventions on Musculoskeletal Pain and Physical Function in Manufacturing Workers: A Systematic Review and Meta-Analysis
Source: Healthcare (Basel). 2026 Oct 1;14(19):3230. doi: 10.3390/healthcare14193230 (PMC13664732; doi:10.3390/healthcare14193230)
Supplement: Supplementary file 1 [file healthcare-14-03230-s001.zip › Supplementary S7.pdf]

Supplementary S7 Summary of findings: Exercise-based interventions compared to control for manufacturing workers with work-related musculoskeletal disorders

Certainty assessment							№ of patients		Effect		Certainty	Importance
**№ of studies**	**Study design**	**Risk of bias**	**Inconsistency**	**Indirectness**	**Imprecision**	**Other considerations**	**Exercise-based interventions**	**Control**	**Relative (95% CI)**	**Standardized mean difference (95% CI)**		
Pain intensity (follow-up: range 8 weeks to 10 months; assessed with: NRS, VAS, and the AUSCAN pain subscale)												
7	Randomized trials	serious[a]	serious[b]	not serious	serious[c]	See note j	204	210	-	−0.69 (−1.25 to −0.12)	⊕◯◯◯ Very low[a,b,c]	CRITICAL
Disability/activity limitation (AUSCAN physical function, RMDQ, ODI)												
3	Randomized trials	Serious[d]	Not serious	Not serious	Serious[e]	See note j	74	69		−0.67 (−1.42 to 0.07)	⊕⊕◯◯ Low[d,e]	
Shoulder abduction range of motion												
2	Randomized trials	Serious[f]	Not serious	Not serious	Very serious[g]	See note j	47	64		−0.63 (−3.29 to 2.03)	⊕◯◯◯ Very low[f,g]	
RAND-36 physical functioning												
1	Randomized trial	Serious[h]	Not assessable	Not serious	Very serious[i]	See note j	16	8		−0.21 (−1.06 to 0.64)	⊕◯◯◯ Very low[h,i]	

CI: confidence interval; SMD: standardised mean difference; NRS: numerical rating scale; VAS: visual analog scale; AUSCAN: Australian/Canadian Osteoarthritis Hand Index.

a. Risk of bias: Downgraded by one level because contributing studies had concerns regarding deviations from intended interventions and, in some studies, outcome measurement or missing outcome data. For example, the reassessment of Nassif et al. identified concerns about incomplete outcome data and a high risk of bias in the measurement of self-reported pain. Lack of participant or provider blinding alone was not treated as sufficient justification for downgrading.

b. Inconsistency: Downgraded by one level because the revised pain synthesis showed substantial statistical heterogeneity (I^2 = 71.9%), with variation in the magnitude of study effects. Differences in occupational exposures, anatomical regions, intervention content, and comparators are plausible contributors, but their individual contributions were not established.

c. Imprecision: Downgraded by one level because the 95% confidence interval for the pooled SMD ranged from −1.25 to −0.12, encompassing markedly different magnitudes of benefit. Although the interval excluded no effect, it did not provide a precise estimate of the benefit. No common instrument-specific threshold for a clinically important difference was established across the pooled pain measures.

d. Risk of bias: Downgraded by one level because the contributing evidence included concerns about self-reported outcome measurement and incomplete outcome data, particularly in Nassif et al. Lack of blinding alone was not considered sufficient justification for downgrading.

e. Imprecision: Downgraded by one level because the evidence comprised three studies with 143 participants, and the confidence interval included both a substantial benefit and little or no difference (SMD −0.67, 95% CI −1.42 to 0.07).

f. Risk of bias: Downgraded by one level because the metalworker trial by Rasotto et al. excluded intervention participants after randomization, including participants with low attendance. Only 17 of 34 participants allocated to exercise were included in the analysis, compared with 34 of 34 controls. This raises concerns about bias arising from post-randomization exclusions.

g. Imprecision: Downgraded by two levels because only two studies with 111 participants contributed data, and the confidence interval encompassed large effects in both directions (SMD −0.63, 95% CI −3.29 to 2.03).

h. Risk of bias: Downgraded by one level because the single contributing trial provided insufficient detail about random-sequence generation and allocation concealment, and the outcome was self-reported in a setting where the exercise interventions were apparent to participants.

i. Imprecision: Downgraded by two levels because the evidence came from one trial with 24 participants and the confidence interval included appreciable benefit and harm (SMD −0.21, 95% CI −1.06 to 0.64).

j. Additional considerations: No additional downgrade was applied for publication bias, but publication bias could not be excluded. The small number of studies limited its assessment. No downgrade for inconsistency was applied to the disability or range-of-motion analyses, although the small number of studies limits confidence in heterogeneity estimates. Inconsistency could not be assessed for the single-study RAND-36 result. Negative SMDs favour exercise after harmonization of outcome direction.
