# Supplementary material for: Effects of Exercise-Based Interventions on Musculoskeletal Pain and Physical Function in Manufacturing Workers: A Systematic Review and Meta-Analysis
Source: Healthcare (Basel). 2026 Oct 1;14(19):3230. doi: 10.3390/healthcare14193230 (PMC13664732; doi:10.3390/healthcare14193230)
Supplement: Supplementary file 1 [file healthcare-14-03230-s001.zip › Supplementary S8.pdf]

Supplementary S8. PRISMA 2020 Checklist

Locations refer to the final proof manuscript (healthcare-4523760). Section, figure, table and supplementary-file identifiers are used so that locations remain usable after final pagination.

Item	PRISMA 2020 checklist item	Location in manuscript / reporting note
1	Identify the report as a systematic review.	Title page.
2	See the PRISMA 2020 for Abstracts checklist.	Abstract.
3	Describe the rationale for the review in the context of existing knowledge.	Section 1 (Introduction).
4	Provide an explicit statement of the objective(s) or question(s) the review addresses.	Section 1, final paragraph.
5	Specify the inclusion and exclusion criteria for the review and how studies were grouped for the syntheses.	Section 2.1 (eligibility criteria); Section 2.4 (outcome-domain groupings and eligibility for each synthesis).
6	Specify all databases, registers, websites, organisations, reference lists and other sources searched or consulted to identify studies. Specify the date when each source was last searched or consulted.	Section 2.1, first paragraph. Search coverage through 31 March 2026 is given; database-specific execution dates are not documented.
7	Present the full search strategies for all databases, registers and websites, including any filters and limits used.	Supplementary S1; Section 2.1. Platforms and additional search limits are not fully documented.
8	Specify the methods used to decide whether a study met the inclusion criteria of the review, including how many reviewers screened each record and each report retrieved, whether they worked independently, and if applicable, details of automation tools used in the process.	Section 2.1, final paragraph (independent screening and resolution of disagreements).
9	Specify the methods used to collect data from reports, including how many reviewers collected data from each report, whether they worked independently, any processes for obtaining or confirming data from study investigators, and if applicable, details of automation tools used in the process.	Section 2.1, final paragraph (data extraction).
10a	List and define all outcomes for which data were sought. Specify whether all results that were compatible with each outcome domain in each study were sought (e.g. for all measures, time points, analyses), and if not, the methods used to decide which results to collect.	Sections 2.1 and 2.4. Outcomes, domains, time points and selected comparisons are described; selection details are partial.
10b	List and define all other variables for which data were sought (e.g. participant and intervention characteristics, funding sources). Describe any assumptions made about any missing or unclear information.	Section 2.1, final paragraph; Section 3.1; Table 1.
11	Specify the methods used to assess risk of bias in the included studies, including details of the tool(s) used, how many reviewers assessed each study and whether they worked independently, and if applicable, details of automation tools used in the process.	Section 2.2; Section 3.2; Supplementary S2-S4. A complete outcome-specific reassessment was not available.
12	Specify for each outcome the effect measure(s) (e.g. risk ratio, mean difference) used in the synthesis or presentation of results.	Section 2.4 (Hedges’ g and effect direction).

Item	PRISMA 2020 checklist item	Location in manuscript / reporting note
13a	Describe the processes used to decide which studies were eligible for each synthesis (e.g. tabulating the study intervention characteristics and comparing against the planned groups for each synthesis (item #5)).	Section 2.4; Section 3.3 (studies contributing to each synthesis).
13b	Describe any methods required to prepare the data for presentation or synthesis, such as handling of missing summary statistics, or data conversions.	Section 2.4 (change scores, imputed variability, combined exercise arms and source-data discrepancies).
13c	Describe any methods used to tabulate or visually display results of individual studies and syntheses.	Section 3.3; Figures 2 and 3 and their captions.
13d	Describe any methods used to synthesize results and provide a rationale for the choice(s). If meta-analysis was performed, describe the model(s), method(s) to identify the presence and extent of statistical heterogeneity, and software package(s) used.	Section 2.4 (software, REML, modified Hartung-Knapp intervals and heterogeneity statistics).
13e	Describe any methods used to explore possible causes of heterogeneity among study results (e.g. subgroup analysis, meta-regression).	Section 2.4, final paragraph; Sections 3.4 and 3.5. Formal subgroup and moderator analyses were not retained.
13f	Describe any sensitivity analyses conducted to assess robustness of the synthesized results.	Section 2.4 (source-data and correlation sensitivity analyses; leave-one-out methods); Section 3.7.
14	Describe any methods used to assess risk of bias due to missing results in a synthesis (arising from reporting biases).	Section 2.4, final paragraph; Section 3.6. Formal asymmetry tests and trim-and-fill analyses were not retained.
15	Describe any methods used to assess certainty (or confidence) in the body of evidence for an outcome.	Section 2.3 (Certainty of Evidence Assessment).
16a	Describe the results of the search and selection process, from the number of records identified in the search to the number of studies included in the review, ideally using a flow diagram.	Section 3.1, first two paragraphs; Figure 1.
16b	Cite studies that might appear to meet the inclusion criteria, but which were excluded, and explain why they were excluded.	Section 3.1, first paragraph; Figure 1. Exclusion counts and categories are given; study-specific exclusion citations are not provided.
17	Cite each included study and present its characteristics.	Section 3.1; Table 1.
18	Present assessments of risk of bias for each included study.	Section 3.2; Supplementary S2-S4. Lowe et al. is discussed narratively; a complete cluster-specific assessment was not available.
19	For all outcomes, present, for each study: (a) summary statistics for each group (where appropriate) and (b) an effect estimate and its precision (e.g. confidence/credible interval), ideally using structured tables or plots.	Section 3.3; Figures 2 and 3. Arm-level summary statistics are not fully tabulated.
20a	For each synthesis, briefly summarise the characteristics and risk of bias among contributing studies.	Sections 3.1-3.3; Table 1; Supplementary S2-S4 (partial; report-based assessments).
20b	Present results of all statistical syntheses conducted. If meta-analysis was done, present for each the summary estimate and its precision (e.g. confidence/credible interval) and measures of statistical heterogeneity. If comparing groups, describe the direction of the effect.	Sections 3.3 and 3.7; Figures 2 and 3; Supplementary S5 and S6.

Item	PRISMA 2020 checklist item	Location in manuscript / reporting note
20c	Present results of all investigations of possible causes of heterogeneity among study results.	Sections 3.4 and 3.5. Formal subgroup and moderator analyses were not retained.
20d	Present results of all sensitivity analyses conducted to assess the robustness of the synthesized results.	Section 3.7; Supplementary S5 and S6.
21	Present assessments of risk of bias due to missing results (arising from reporting biases) for each synthesis assessed.	Section 3.6. Publication bias cannot be excluded.
22	Present assessments of certainty (or confidence) in the body of evidence for each outcome assessed.	Section 3.8; Supplementary S7.
23a	Provide a general interpretation of the results in the context of other evidence.	Section 4 (Discussion), interpretation and comparison with previous reviews.
23b	Discuss any limitations of the evidence included in the review.	Section 4, limitations paragraphs (clinical diversity, study limitations and imprecision).
23c	Discuss any limitations of the review processes used.	Section 4, review-process limitations (search restrictions, analytical assumptions and unavailable analyses).
23d	Discuss implications of the results for practice, policy, and future research.	Section 4, clinical implications and future research; Section 5 (Conclusions).
24a	Provide registration information for the review, including register name and registration number, or state that the review was not registered.	Abstract, Methods; Section 2.1, first paragraph: PROSPERO CRD420261369113.
24b	Indicate where the review protocol can be accessed, or state that a protocol was not prepared.	Section 2.1 provides the registration identifier. Separate protocol access is not reported.
24c	Describe and explain any amendments to information provided at registration or in the protocol.	Section 2.4 describes analyses and domain groupings introduced during revision and identifies them as not prespecified.
25	Describe sources of financial or non-financial support for the review, and the role of the funders or sponsors in the review.	Funding statement. Funding sources and grant number are given; the funders' role is not reported.
26	Declare any competing interests of review authors.	Conflicts of Interest statement.
27	Report which of the following are publicly available and where they can be found: template data collection forms; data extracted from included studies; data used for all analyses; analytic code; any other materials used in the review.	Data Availability Statement. Published source reports are identified; public access to extraction data and code is not specified.

Source: Page MJ, McKenzie JE, Bossuyt PM, Boutron I, Hoffmann TC, Mulrow CD, et al. The PRISMA 2020 statement: an updated guideline for reporting systematic reviews. BMJ. 2021;372:n71. https://doi.org/10.1136/bmj.n71. Listed as Reference [24] in the manuscript.

Adapted from the PRISMA 2020 checklist, licensed under CC BY 4.0: https://creativecommons.org/licenses/by/4.0/. Checklist items are retained; the layout and manuscript-location column have been updated. S = Supplementary.
